# Cataract Services are Leaving Widows Behind: Examples from National Cross-Sectional Surveys in Nigeria and Sri Lanka

**DOI:** 10.3390/ijerph16203854

**Published:** 2019-10-12

**Authors:** Jacqueline Ramke, Fatima Kyari, Nyawira Mwangi, MMPN Piyasena, GVS Murthy, Clare E Gilbert

**Affiliations:** 1 International Centre for Eye Health, London School of Hygiene and Tropical Medicine, London WC1E 7HT, UK; nyawira.mwangi@lshtm.ac.uk (N.M.); prabhath.nishantha@gmail.com (M.P.); GVS.Murthy@lshtm.ac.uk (G.M.); clare.gilbert@lshtm.ac.uk (C.E.G.); 2 School of Optometry and Vision Science, University of Auckland, Auckland 1010, New Zealand; 3 College of Health Sciences, Baze University, Abuja 900108, Nigeria; fatima.kyari@bazeuniversity.edu.ng; 4 Department of Clinical Medicine, Kenya Medical Training College, Nairobi 00100, Kenya; 5 Ministry of Health, Indigenous Medicine and Nutrition, Policy Analysis and Development Unit, Colombo 10, Sri Lanka; 6 Public Health Foundation of India, Hyderabad, Telangana 122002, India

**Keywords:** health equity, health inequality, universal eye health, universal health coverage, effective cataract surgical coverage, cataract services, healthy aging, widowhood

## Abstract

The Sustainable Development Goals aim to leave no one behind. We explored the hypothesis that women without a living spouse—including those who are widowed, divorced, separated, and never married—are a vulnerable group being left behind by cataract services. Using national cross-sectional blindness surveys from Nigeria (2005–2007; *n* = 13,591) and Sri Lanka (2012–2014; *n* = 5779) we categorized women and men by marital status (married/not-married) and place of residence (urban/rural) concurrently. For each of the eight subgroups we calculated cataract blindness, cataract surgical coverage (CSC), and effective cataract surgical coverage (eCSC). Not-married women, who were predominantly widows, experienced disproportionate cataract blindness—in Nigeria they were 19% of the population yet represented 56% of those with cataract blindness; in Sri Lanka they were 18% of the population and accounted for 54% of those with cataract blindness. Not-married rural women fared worst in access to services—in Nigeria their CSC of 25.2% (95% confidence interval, CI 17.8–33.8%) was far lower than the best-off subgroup (married urban men, CSC 80.0% 95% CI 56.3–94.3); in Sri Lanka they also lagged behind (CSC 68.5% 95% CI 56.6–78.9 compared to 100% in the best-off subgroup). Service quality was also comparably poor for rural not-married women—eCSC was 8.9% (95% CI 4.5–15.4) in Nigeria and 37.0% (95% CI 26.0–49.1) in Sri Lanka. Women who are not married are a vulnerable group who experience poor access to cataract services and high cataract blindness. To “leave no one behind”, multi-faceted strategies are needed to address their needs.

## 1. Introduction

In 2015 most governments of the world signed the United Nation’s Sustainable Development Goals (SDGs), which make a fundamental commitment to “leave no one behind” and include a goal to “ensure healthy lives and promote wellbeing for all at all ages” [1]. To translate the SDGs into effective action for health, population subgroups who experience the worst health need to be identified and strategies adopted to meet the needs of those who are furthest behind.

In terms of health, women are disproportionately found among those who have been left behind. Throughout the world women experience overt and unconscious bias from their households, communities, and societies, reducing their ability to exercise their right to health [2]. Certain subgroups of women are particularly vulnerable to poor health in different contexts, such as women from poorer households, and those marginalized due to their marital status, age, or physical ability [3].

For eye health, the experiences of older women are particularly relevant, with cataract being a leading cause of reduced functional ability with aging [4,5]. Women are more likely than men to develop some types of cataract [6], but over and above biological causes, the higher prevalence of cataract blindness in women [7] is likely due to women experiencing higher exposure to known risk factors than men, such as biomass cooking [8] and childbearing [9,10], as well as lower access to [11] and poorer quality of [12] cataract surgical services.

Beyond biological changes, older age also involves changes in roles and social position, including increased reliance on adult children [4]. In many low- and middle-income countries, elderly people undergoing cataract surgery rely on social support from their children to access services, and in studies in India [13] and Tanzania [14], women have reported more difficulty than men to negotiate this family support. Social support is rarely measured in eye health research. Marriage is the primary social relationship for many adults, and while being married is not universally beneficial to health, married people are assumed to have higher levels of support and material resources than people without a spouse [3,15]. In this study we define not-married as being without a living spouse, and include those who were widowed, divorced, separated, and never married. Due to longer life expectancy, women are more likely than men to be widowed, and in some societies are less likely to remarry than widowed men [3]. Therefore, women are more likely than men to experience the social and health consequences of widowhood. Indeed, a recent community survey of cataract blind people in Nigeria found 53% of women blind from cataract were not married, compared to only 6% of men blind from cataract [16].

The aim of this study was to explore the hypothesis that not-married women experience a disproportionate amount of cataract blindness and poorer cataract services indicators than other population subgroups.

## 2. Materials and Methods

### 2.1. Data Source

Cross-sectional data from nationwide representative surveys of adults aged ≥40 years undertaken in Nigeria (2005–2007; *n* = 13,591) and Sri Lanka (2012–2014; *n* = 5779) were used in this analysis. These surveys were chosen as they used a similar methodology, collected comparable social variables, and are among the few national-level visual impairment surveys conducted in the past 15 years that collected information on marital status. The methods have been described in detail [17,18]. In both countries cataract blindness (best-corrected vision) was higher in women than men (Nigeria: 1.9% (95% confidence interval, CI 1.6–2.3) in women and 1.1% (95% CI 0.9–1.4) in men [19]; Sri Lanka: 1.0% (95% CI 0.7–1.4) in women and 0.8% (95% CI 0.5–1.3) in men) [18].

Ethical approval for the surveys was granted prior to original collection by the ethics committee at London School of Hygiene & Tropical Medicine (Nigeria 29/9/2004, Ref2040; Sri Lanka 18/4/2012 Ref6389) and the relevant entities in each country.

### 2.2. Variables

The following descriptive outcome variables were used:

1. *Cataract blindness*, defined as best-corrected visual acuity of <3/60 in the better eye where the principal cause was cataract;
2. *Cataract surgical coverage* (CSC), which measures the number of people in a defined population with operated cataract as a proportion of those having operable plus operated cataract. We defined “operable cataract” as a cataract causing best-corrected visual acuity worse than 3/60; and
3. *Effective cataract surgical coverage* (eCSC), which measures the number of people in a defined population with operated cataract and a good outcome (i.e., presenting visual acuity 6/18 or better) as a proportion of those having operable plus operated cataract [12].

Explanatory variables were marital status (married versus not-married (widowed, divorced, separated or never married)), and area of residence (urban versus rural). We included area of residence in addition to marital status, as urban dwellers often experience better access to services. We did not disaggregate further by socio-economic status, as subgroups became too small.

### 2.3. Analysis

The proportion of participants who were not-married was calculated for women and men in each country, and the distribution of marital status across 10-year age groups of women and men was plotted.

Women and men were disaggregated by marital status and area of residence simultaneously, and cataract blindness, CSC, eCSC, and 95% CIs were calculated for the eight subgroups generated (i.e., urban married, urban not-married, rural married, rural not-married, for both women and men) (Supplemental Table S1).

The social distribution of cataract blindness across the eight subgroups was examined using bar graphs, with bar width reflecting the proportion of the sample in each subgroup. Finally, CSC and eCSC and their 95% CIs for the eight subgroups were plotted for each country.

Analyses were conducted using STATA 12.0 (StataCorp, College Station, TX, USA). Statistical significance was implied by 95% confidence intervals which did not overlap.

## 3. Results

### 3.1. Marital Status

Women were more likely to be not-married than men—fewer than 1 in 10 men in Sri Lanka (8.5%) and fewer than 1 in 20 men in Nigeria (4.8%) were not-married compared to approximately 1 in 3 women in both Nigeria (34.4%) and Sri Lanka (30.2%). Most not-married women were widows (95% in Nigeria and 82% in Sri Lanka).

**Figure 1:**
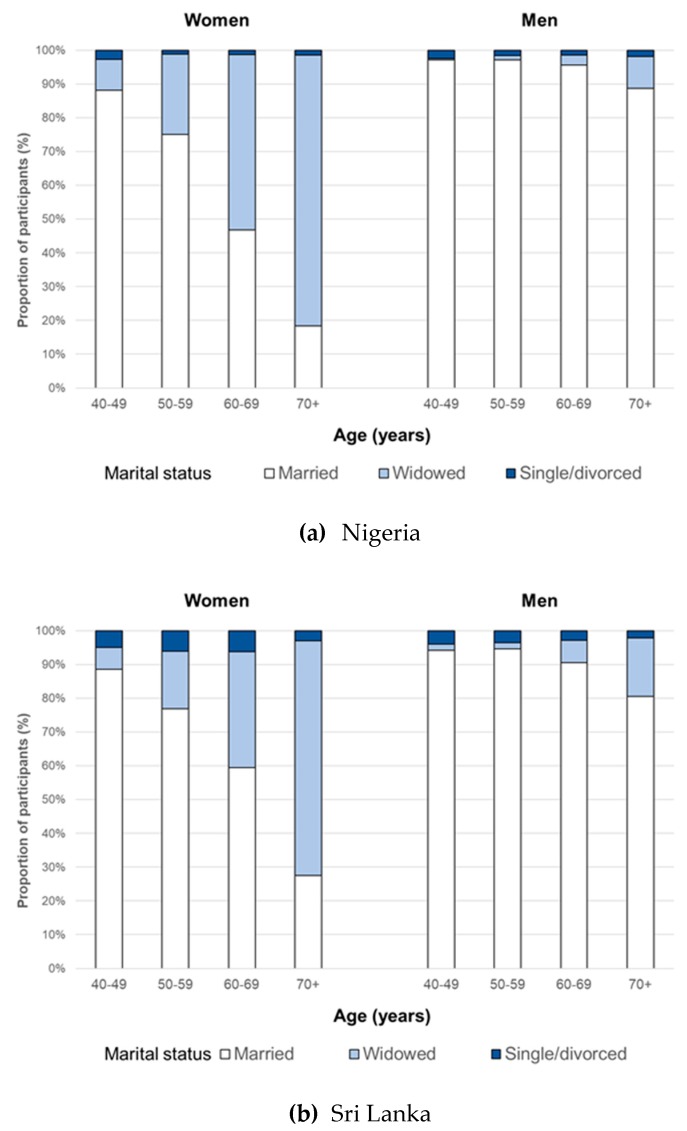
Marital status in women and men across age groups in (**a**) Nigeria (2005–2007) and (**b**) Sri Lanka (2012–2014).

The distribution of marital status across age groups was similar in the two countries. Most women 60 years or older were not-married in Nigeria (65.7%) and Sri Lanka (52.1%), increasing to 81.7% and 72.5% for women aged 70 years and above. The corresponding figures for men were 7.7%/11.4% (≥60 years), and 13.0%/19.5% (≥70 years) in Nigeria and Sri Lanka, respectively (Figure 1).

### 3.2. Cataract Blindness

**Figure 2:**
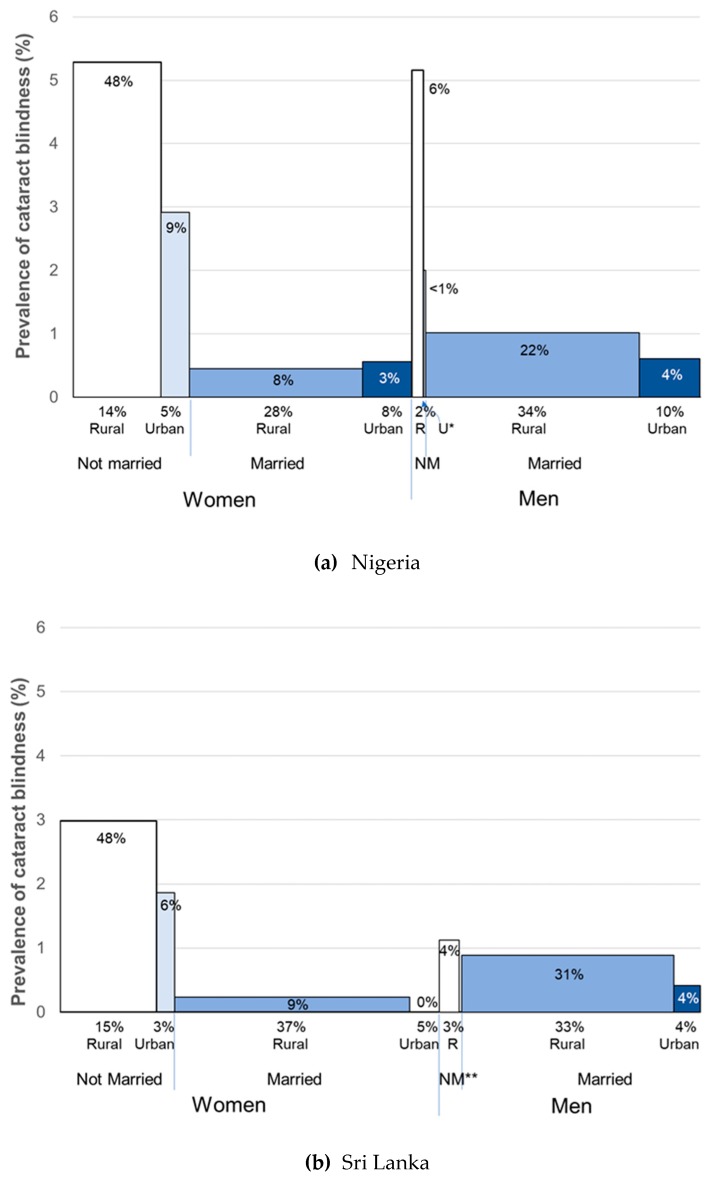
Prevalence of cataract blindness across social subgroups of women and men aged ≥40 years in (**a**) Nigeria (2005–2007) and (**b**) Sri Lanka (2012–2014). For each subgroup, the prevalence of cataract blindness is plotted along the y axis, and the proportion of the sample in each subgroup is indicated under the *x*-axis. For example, in Nigeria 14% of the sample were rural not-married women. The number above each bar indicates the proportion of all cataract blindness in the subgroup, for example, in Nigeria 48% of the cataract blind were rural not-married women. R = rural, U = urban, NM = not-married, * = very few urban not-married men in Nigeria, ** = zero urban not-married men with cataract blindness in Sri Lanka.

Women comprised approximately two-thirds of the cataract blind in both countries (67% in Nigeria and 63% in Sri Lanka) (Figure 2). Not-married women experienced cataract blindness disproportionately—in Nigeria they represented 19% of the survey sample and 56% of the cataract blind, and in Sri Lanka they accounted for 18% of the sample and 54% of the cataract blind. Rural not-married were the subgroup of men with the highest rate of cataract blindness, but due to there being so few of them, they represented only 6% of the cataract blind in Nigeria, and 4% in Sri Lanka (Figure 2).

### 3.3. Cataract Services

**Figure 3:**
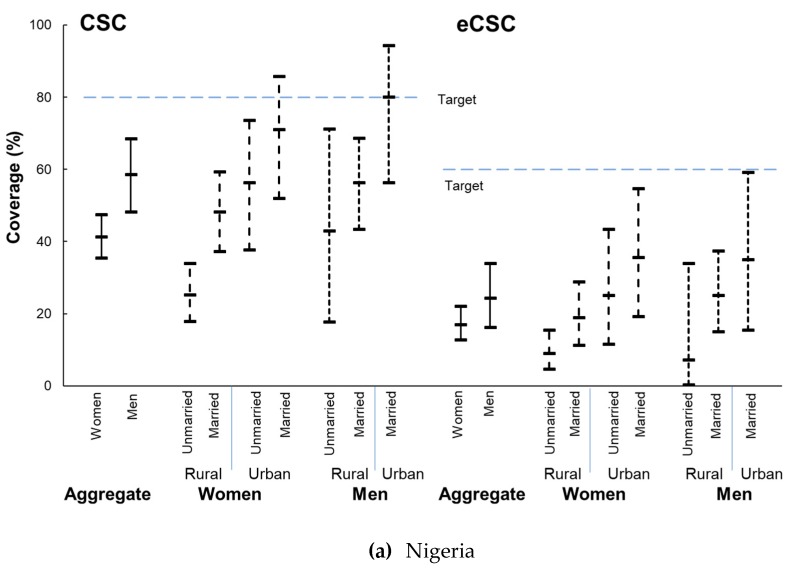

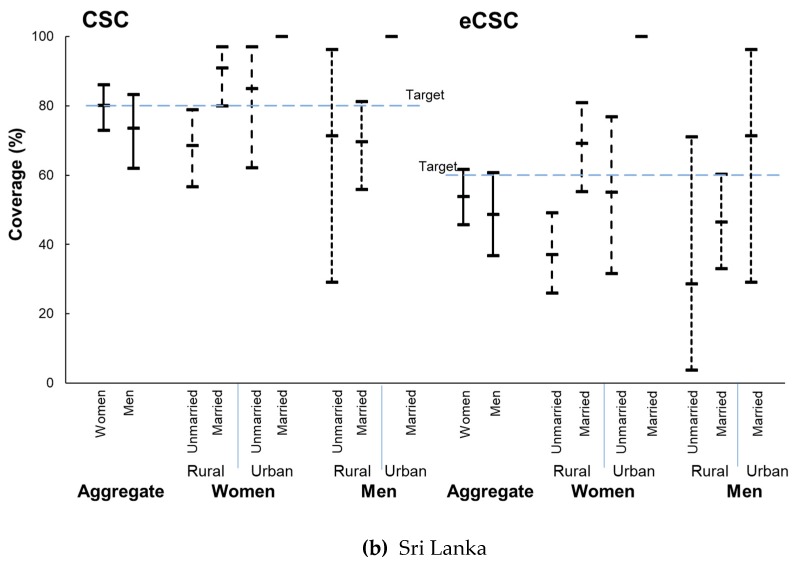
Cataract surgical coverage (CSC) and effective cataract surgical coverage (eCSC) across social subgroups of women and men aged ≥40 years in (**a**) Nigeria (2005–2007) and (**b**) Sri Lanka (2012–2014). CSC, eCSC, and their 95% CI are shown by the vertical bar for each subgroup. CSC target 80% [20], eCSC target 60% [21]. The CSC value in married urban women and men and urban not-married men in Sri Lanka was 100%, as was eCSC in urban not-married men. In Nigeria, zero not-married urban men had surgery.

At the aggregate level, only women in Sri Lanka met global CSC (>80%) or eCSC (>60%) [12] targets. When disaggregated into subgroups, married women (urban and rural) and married urban men in Sri Lanka met both CSC and eCSC targets (Figure 3).

A gradient in CSC and eCSC was observed in both countries, with rural dwellers tending to fare worse than urban dwellers, and within each area of residence, those who were not-married fared worse than those who were married. One exception was in Sri Lanka, where urban not-married women fared worse than rural married women. Rural not-married women and men had the lowest coverage and urban married women and men had the highest (Figure 3).

In Nigeria the difference between the best- and worst-off subgroups of women was greater than the aggregate gap between women and men for both outcomes—CSC and eCSC values in rural not-married women were 45.8% and 26.6% less than rates in urban married women, with differences between women and men of 17.3% for CSC and 7.2% for eCSC (Figure 3, data shown in Supplemental Table S1).

In Sri Lanka women had slightly better CSC and eCSC than men at the aggregate level (CSC in women 80.1%; 95% CI 73.0–86.1 compared to men 73.6%; 95% CI 61.9–83.3). Findings for eCSC were similar (in women 53.8%; 95% CI 45.7–61.6 compared to 48.6%; 95% CI 36.7–60.7 in men). Married women fared as well or better than their male counterparts (e.g., CSC and eCSC in rural married women were better than in rural married men) (Figure 3). The gap between women and men was 6.5% for CSC and 5.2% for eCSC, but confidence intervals overlapped (data shown in Supplemental Table S1). However, the difference between urban married women and rural not-married women was much larger—31.5% for CSC and 63.0% for eCSC (Figure 3, data shown in Supplemental Table S1).

## 4. Discussion

Despite the different cultural and health system contexts of these two countries, and the 10 years between surveys, in both settings women and men without a spouse experienced a disproportionate level of cataract blindness (Figure 2) and worse service access and quality (Figure 3) compared to married women and men.

Higher cataract blindness prevalence in the not-married is unsurprising when the age distribution of marital status is considered—just as cataract blindness increases with older age [5], so too did being not-married in both Nigeria and Sri Lanka, particularly widowhood, and to a much greater extent for women than men (Figure 1). Rural men without a spouse fared as poorly as their female counterparts, but they were a much smaller portion of the population—2% of the sample in Nigeria and 3% in Sri Lanka were rural not-married men compared to 14% and 15% being rural not-married women in Nigeria and Sri Lanka, respectively (Figure 2). The number of urban not-married men was so small CSC and eCSC results could not be calculated.

Cataract provides an example of the increased health care needs in ageing populations, and by helping to maintain functional ability, effective cataract surgery is an important health care component of healthy ageing [4]. Indeed, a recent analysis in the United States found that among older women, cataract surgery was associated with decreased risk for all-cause mortality and risk related to multiple types of systemic illness [22]. CSC and eCSC (Figure 3) reflect access and quality of cataract services only among those requiring care, so are less confounded by age than cataract blindness. Nevertheless, the not-married continued to experience worse outcomes compared to their married counterparts in urban and rural areas, and the rural not-married fared worst of all. To reduce inequality in cataract blindness in these countries and “leave no one behind”, the social distribution of blindness should be reflected in the social distribution of those who access cataract surgery. In these settings, women without a spouse must be at least half of those undergoing cataract surgery to avoid them being left further behind.

The lower access and worse quality of services experienced by rural dwellers in both settings highlight that eye health services are more readily available and better quality in urban centers [23,24,25]. Where outreach services are insufficient, rural dwellers must travel to access care. Even if surgical costs are subsidized—or fully funded as in Sri Lanka—the non-medical costs to patients are substantial. For example, in a recent study in a rural area of Nigeria the median direct cost of undergoing surgery for those living further than 50 kilometers (km) from the hospital was US$84, more than twice that for those living within 50 km [26]. In the same study 92% of men had sufficient funds to pay for surgery, with the remaining 8% having to sell assets or take out loans. Married women sold assets or took a loan at a similar rate to men (7%), while a higher proportion of unmarried women (20%) had raised funds through selling assets or taking out a loan [26].

The distribution of marital status across age groups (Figure 1) and the distribution of CSC and eCSC across social subgroups (Figure 3) were similar in these two very different social contexts, suggesting those without a spouse were unable to exercise their right to health to the same extent as married people. Further, the findings suggest that the role of social support for women and men in accessing cataract services warrants greater attention, with emphasis on the barriers experienced by not-married cataract blind women in rural areas.

Lack of social support for women to access cataract services is a recognized problem in Africa [27], but the role of marital status is not homogeneous. In contrast to our findings, a study in Malawi found widowed women were more likely to have surgery than married women [28], suggesting that in some contexts being married does not promote health. Indeed, women in Tanzania reported that their husbands could be an additional barrier to undergoing cataract surgery [14]. These contrasting findings highlight that generalizations cannot be made between settings, but social support is arguably an important determinant of access to good quality care in many settings, and should be considered when planning equitable services.

Cataract services must redress existing inequalities in the context of an ageing population, urbanization, and changes to family structures and intergenerational relationships, in which elderly people often have diminished authority [4]. In settings where the elderly rely on their children to access cataract surgery, demographic changes result in fewer children to support their parents, with the likelihood of doing so decreasing [14]. Given their longer life expectancy and lower social status, women are more vulnerable to these social changes compared to men [3], suggesting that the worse outcomes among not-married women we observed could increase in future.

The social changes occurring throughout the world indicate that families, policy-makers and health services must identify new solutions to ensure older women—many of whom are widowed—can age healthily [3]. For cataract services, this could involve implementing and evaluating combinations of targeted strategies shown to work in other settings, including providing good quality services nearer to where people live, provision of transport to and from hospital, use of a motivator, and elimination of surgical fees [27,29,30,31]. To gain more social support it may help to strengthen counselling for blind women and their families, and highlight the benefits to men of their wives and mothers undergoing surgery [32].

The growing number of older women could be considered a societal resource, whose health and functional ability is valued. Given the key caregiver role older women have in their families and communities [33,34], extending and enhancing their lives should be seen as sound economic and social sense rather than a burden [3]. Global initiatives such as the promotion of health rights, the SDGs, and Universal Health Coverage all provide opportunities to promote better health, and better cataract services, for vulnerable older women.

Our findings reinforce the need to look beyond aggregate indicators [35] when planning services so that planners can see which subgroups in the population are most in need and then develop more refined strategies to reach these groups. For example, in Sri Lanka women overall had slightly better aggregate service indicators (CSC and eCSC) than men. This aggregate-level gender-equity is positive, but it obscured the poorer access and quality of cataract surgery experienced by rural women without a spouse (Figure 3). In Nigeria, improving uptake of services by rural not-married women would markedly reduce the disparity in cataract blindness between women and men. Targeting specific groups also allows resources to be more efficiently allocated—for example, in Sri Lanka urban women and men met service targets, so efforts should focus on maintaining services in urban centers while improving access and quality of rural services.

An indicator of social support is not routinely collected in eye health research and in this analysis we used marital status as a proxy for social support. Other social support indicators that could be relevant to explore social support and cataract services include relationship with household head, contribution to household finances, number of living children, receipt of help with daily activities, and involvement in household health decisions [36]. More in-depth qualitative analysis in specific contexts is needed to understand how to improve social support for the vulnerable to undergo cataract surgery [14]. In addition, to assist monitoring, there may be a place for broader collection of key social support variables in common tools such as the Rapid Assessment of Avoidable Blindness (RAAB).

These results must be interpreted in the context of several limitations. First, we recognize that the not-married group is not homogenous. Those who were divorced, separated, or single tended to be younger than those who were widowed (Figure 1), and thus had lower rates of cataract blindness. However, the number of non-widows was small—particularly in Nigeria—so the influence of this group on the overall results was minimal. Second, we did not explore socioeconomic status concurrently with place of residence and marital status, as our group sizes became very small. It is conceivable that disadvantage would accumulate [21] such that those who are poorer in addition to living rurally and being without a spouse experienced even worse access to cataract services. Third, the surveys—particularly Nigeria—were taken some time ago. Therefore, the findings do not necessarily reflect the current situation in these countries, though we are unaware of strategies being implemented that may have mitigated the inequity identified here. Finally, while not strictly a limitation, we did not age-adjust our results, as our aim was to determine the distribution of cataract blindness across the population subgroups and therefore the groups being left behind.

## 5. Conclusions

We have explored the distribution of cataract blindness and cataract service outcomes across sex, marital status, and area of residence, and identified that more support is indicated for rural people without a spouse—many more of whom are women—if equity is to be achieved. We urge eye health researchers, service providers, and planners to collect and monitor indicators of social support, including marital status, and then implement targeted interventions to ensure no one is left behind.

## Supplementary Materials

The following are available online at https://www.mdpi.com/1660-4601/16/20/3854/s1, Supplemental Table S1: Cataract blindness, cataract surgical coverage (CSC), and effective cataract surgical coverage (eCSC) in subgroups of women and men aged ≥40 years in Nigeria (2005–2007) and Sri Lanka (2012–2014).

## References

[1] United Nations General Assembly (2015). Transforming our World: The 2030 Agenda for Sustainable Development. Resolution Adopted by the General Assembly on 25 September 2015. A/RES/70/1.

[2] Pratley P. (2016). Associations between quantitative measures of women’s empowerment and access to care and health status for mothers and their children: A systematic review of evidence from the developing world. Soc. Sci. Med..

[3] World Health Organization (2009). Women and Health: Today’s Evidence Tomorrow’s Agenda.

[4] World Health Organization (2015). World Report on Ageing and Health.

[5] Bourne R.R., Stevens G.A., White R.A., Smith J.L., Flaxman S.R., Price H., Jonas J.B., Keeffe J., Leasher J., Naidoo K. (2013). Causes of vision loss worldwide, 1990–2010: A systematic analysis. Lancet Glob. Health.

[6] West S. (2007). Epidemiology of cataract: Accomplishments over 25 years and future directions. Ophthalmic Epidemiol..

[7] Flaxman S., Bourne R., Resnikoff S., Ackland P., Braithwaite T., Cicinelli M.V., Das A., Jonas J.B., Keeffe J., Kempen J.H. (2017). Global Causes of Distance Vision Loss: 1990–2015 and projections to 2020. Lancet Glob. Health.

[8] Ravilla T.D., Gupta S., Ravindran R.D., Vashist P., Krishnan T., Maraini G., Chakravarthy U., Fletcher A.E. (2016). Use of cooking fuels and cataract in a population-based study: The India Eye Disease Study. Environ. Health Perspect..

[9] Minassian D., Mehra V., Reidy A. (2002). Childbearing and risk of cataract in young women: An epidemiological study in central India. Br. J. Ophthalmol..

[10] Tian Y., Wu J., Xu G., Shen L., Yang S., Mandiwa C., Yang H., Liang Y., Wang Y. (2015). Parity and the risk of cataract: A cross-sectional analysis in the Dongfeng-Tongji cohort study. Br. J. Ophthalmol..

[11] Lewallen S., Mousa A., Bassett K., Courtright P. (2009). Cataract surgical coverage remains lower in women. Br. J. Ophthalmol..

[12] Ramke J., Gilbert C., Lee A.C.L., Ackland P., Limburg H., Foster A. (2017). Effective cataract surgical coverage: An indicator for measuring quality-of-care in the context of Universal Health Coverage. PLoS ONE.

[13] Finger R.P., Ali M., Earnest J., Nirmalan P.K. (2007). Cataract surgery in Andhra Pradesh state, India: An investigation into uptake following outreach screening camps. Ophthalmic Epidemiol..

[14] Geneau R., Lewallen S., Bronsard A., Paul I., Courtright P. (2005). The social and family dynamics behind the uptake of cataract surgery: Findings from Kilimanjaro region, Tanzania. Br. J. Ophthalmol..

[15] Wyke S., Ford G. (1992). Competing explanations for associations between marital status and health. Soc. Sci. Med..

[16] Ibrahim N., Ramke J., Pozo-Martin F., Gilbert C.E. (2017). Willingness to pay for cataract surgery is much lower than actual costs in Zamfara state, northern Nigeria. Ophthalmic Epidemiol..

[17] Dineen B., Gilbert C.E., Rabiu M., Kyari F., Mahdi A.M., Abubakar T., Ezelum C.C., Gabriel E., Elhassan E., Abiose A. (2008). The Nigerian National Blindness and Visual Impairment Survey: Rationale, objectives and detailed methodology. BMC Ophthalmol..

[18] Murthy G., Gilbert C., Schmidt E., Mahipala P.G., Gamage K.M.K., Banagala C., Abeydeera A.P., Jeza A. (2018). The Sri Lanka National Blindness, Visual Impairment and Disability Survey: Rationale, objectives and detailed methodology. Ceylon Med. J..

[19] Abdull M.M., Sivasubramaniam S., Murthy G.V.S., Gilbert C., Abubakar T., Ezelum C., Rabiu M.M., Kotecha A., Crabb D.P., Spratt A. (2009). Causes of blindness and visual impairment in Nigeria: The Nigeria National Blindness and Visual Impairment Survey. Investig. Ophthalmol. Vis. Sci..

[20] World Health Organization (1998). Informal Consultation on Analysis of Blindness Prevention Outcomes.

[21] Ramke J., Zwi A.B., Lee A.C., Blignault I., Gilbert C.E. (2017). Inequality in cataract blindness and services: Moving beyond unidimensional analyses of social position. Br. J. Ophthalmol..

[22] Tseng V.L., Chlebowski R.T., Yu F., Cauley J.A., Li W., Thomas F., Virnig B.A., Coleman A.L. (2018). Association of Cataract Surgery with Mortality in Older Women: Findings from the Women’s Health Initiative. JAMA Ophthalmol..

[23] Palmer J.J., Chinanayi F., Gilbert A., Pillay D., Fox S., Jaggernath J., Naidoo K., Graham R., Patel D., Blanchet K. (2014). Mapping human resources for eye health in 21 countries of sub-Saharan Africa: Current progress towards VISION 2020. Hum. Resour. Health.

[24] Estopinal C.B., Ausayakhun S., Ausayakhun S., Jirawison C., Bhosai S.J., Margolis T.P., Keenan J.D. (2013). Access to Ophthalmologic Care in Thailand: A Regional Analysis. Ophthalmic Epidemiol..

[25] Bourne R., Dineen B., Jadoon Z., Lee P.S., Khan A., Johnson G.J., Foster A., Khan D. (2007). Outcomes of cataract surgery in Pakistan: Results from the Pakistan National Blindness and Visual Impairment Survey. Br. J. Ophthalmol..

[26] Ibrahim N., Pozo-Martin F., Gilbert C. (2015). Direct non-medical costs double the total direct costs to patients undergoing cataract surgery in Zamfara state, Northern Nigeria: A case series. BMC Health Serv. Res..

[27] Mganga H., Lewallen S., Courtright P. (2011). Overcoming gender inequity in prevention of blindness and visual impairment in Africa. Middle East Afr. J. Ophthalmol..

[28] Courtright P., Kanjaloti S., Lewallen S. (1995). Barriers to acceptance of cataract surgery among patients presenting to district hospitals in rural Malawi. Trop. Geogr. Med..

[29] Brilliant G.E., Lepkowski J.M., Zurita B., Thulasiraj R.D. (1991). Social determinants of cataract surgery utilization in south India. Arch. Ophthalmol..

[30] Joseph S., Ravilla T., Bassett K. (2013). Gender Issues in a Cataract Surgical Population in South India. Ophthalmic Epidemiol..

[31] Mercer G.D., Lyons P., Bassett K. (2019). Interventions to improve gender equity in eye care in low-middle income countries: A systematic review. Ophthalmic Epidemiol..

[32] Polack S., Eusebio C., Mathenge W., Wadud Z., Rashid M., Foster A., Kuper H. (2010). The impact of cataract surgery on activities and time-use: Results from a longitudinal study in Kenya, Bangladesh and the Philippines. PLoS ONE.

[33] Sear R., Mace R., McGregor I.A. (2000). Maternal grandmothers improve nutritional status and survival of children in rural Gambia. Proc. R. Soc. Lond. B Biol. Sci..

[34] Snopkowski K., Sear R. (2015). Grandparental help in Indonesia is directed preferentially towards needier descendants: A potential confounder when exploring grandparental influences on child health. Soc. Sci. Med..

[35] Östlin P., Schrecker T., Sadana R., Bonnefoy J., Gilson L., Hertzman C., Kelly M.P., Kjellstrom T., Labonté R., Lundberg O. (2011). Priorities for research on equity and health: Towards an equity-focused health research agenda. PLoS Med..

[36] Stagg B.C., Choi H., Woodward M.A., Ehrlich J.R. (2018). Association of Social Support Network Size with Receipt of Cataract Surgery in Older Adults. JAMA Ophthalmol..

