# Supplementary material for: Cataract Services are Leaving Widows Behind: Examples from National Cross-Sectional Surveys in Nigeria and Sri Lanka
Source: Int J Environ Res Public Health. 2019 Oct 12;16(20):3854. doi: 10.3390/ijerph16203854 (PMC6843674; doi:10.3390/ijerph16203854)
Supplement: Supplementary file 1 [file ijerph-16-03854-s001.pdf]

Supplemental Table S1: Cataract blindness, cataract surgical coverage (CSC) and effective cataract surgical coverage (eCSC) in subgroups of women and men aged ≥40 years in Nigeria (2005–7) and Sri Lanka (2012–14)

Country	Subgroup*	n		Cataract blindness % (95%CI)		CSC % (95%CI)		eCSC % (95%CI)	
		Women	Men	Women	Men	Women	Men	Women	Men
Nigeria	Urban Married	1065	1318	0.6 (0.2–1.2)	0.6 (0.3–1.2)	71.0 (52.0–85.8)	80.0 (56.3–94.3)	35.5 (19.2–54.6)	35.0 (15.4–59.2)
	Urban Not-married	618	50	2.9 (1.7–4.6)	2.0 (0.1–10.6)	56.3 (37.7–73.6)	**	25.0 (11.5–43.4)	**
	Rural Married	3750	4624	0.5 (0.3–0.7)	1.0 (0.7–1.3)	48.2 (37.3–59.3)	56.3 (43.3–68.6)	18.8 (11.2–28.8)	25.0 (15.0–37.4)
	Rural Not-married	1911	252	5.3 (4.3–6.4)	5.2 (2.8–8.7)	25.2 (17.8–33.8)	42.9 (17.7–71.1)	8.9 (4.5–15.4)	7.1 (0.2–33.9)
	**Overall**	**7344**	**6244**	**1.9 (1.6–2.3)**	**1.1 (0.9–1.4)**	**41.3 (35.4–47.4)**	**58.6 (48.2–68.4)**	**17.0 (12.7–22.0)**	**24.2 (16.2–33.9)**
Sri Lanka	Urban Married	268	239	0	0.4 (0–2.3)	100	100	100	71.4 (29.0–96.3)
	Urban Not-married	161	23	1.8 (0.4–5.3)	0	85.0 (62.1–97.0)	**	55.0 (31.5–76.9)	**
	Rural Married	2122	1916	0.2 (0.1–0.5)	0.9 (0.6–1.4)	90.9 (80.0–97.0)	69.6 (55.9–81.2)	69.1 (55.2–80.9)	46.4 (33.0–60.3)
	Rural Not-married	872	178	3.0 (2.0–4.3)	1.1 (0.1–4.0)	68.5 (56.6–78.9)	71.4 (29.0–96.3)	37.0 (26.0–49.1)	28.6 (3.7–71.0)
	**Overall**	**3423**	**2356**	**1.0 (0.7–1.4)**	**0.9 (0.5–1.3)**	**80.1 (73.0–86.1)**	**73.6 (61.9–83.3)**	**53.8 (45.7–61.6)**	**48.6 (36.7–60.7)**

*Not-married includes: widowed, divorced, separated or single
**Too few not-married urban men to include results
CSC= number of people with operated cataract as a proportion of those having operable plus operated cataract; operate cataract = best-corrected visual acuity worse than 3/60;
eCSC= number of people with operated cataract and a good outcome (i.e. presenting VA 6/18 or better) as a proportion of those having operable plus operated cataract.
